# Identification of ryuvidine as a KDM5A inhibitor

**DOI:** 10.1038/s41598-019-46346-x

**Published:** 2019-07-09

**Authors:** Eishin Mitsui, Shogo Yoshida, Yui Shinoda, Yasumasa Matsumori, Hiroshi Tsujii, Mie Tsuchida, Shuichi Wada, Makoto Hasegawa, Akihiro Ito, Koshiki Mino, Tetsuo Onuki, Minoru Yoshida, Ryuzo Sasaki, Tamio Mizukami

**Affiliations:** 1 Nagahama Inst. Bio-Sci. & Tech., 1266 Tamura, Nagahama, Shiga, 526-0829 Japan; 2 Sch. of Life Sci., Tokyo Univ. of Pharm. & Life Sci., 1432-1 Horinouchi, Hachioji, Tokyo, 192-0392 Japan; 3 Chem. Genomics, RIKEN CSRS, 2-1 Hirosawa, Wako, Saitama, 351-0198 Japan; 4 Drug Discov. Seed Cpds. Explorat. Unit, RIKEN CSRS, 2-1 Hirosawa, Wako, Saitama, 351-0198 Japan; 5 Department of Biotechnol., The Univ. of Tokyo, 1-1-1 Yayoi, Bunkyo-ku, Tokyo, 113-8657 Japan; 6 Frontier Pharma Inc., 1281-8 Tamura, Nagahama, Shiga, 526-0829 Japan

**Keywords:** High-throughput screening, Targeted therapies

## Abstract

KDM5 family members (A, B, C and D) that demethylate H3K4me3 have been shown to be involved in human cancers. Here we performed screening for KDM5A inhibitors from chemical libraries using the AlphaScreen method and identified a battery of screening hits that inhibited recombinant KDM5A. These compounds were further subjected to cell-based screening using a reporter gene that responded to KDM5A inhibition and 6 compounds were obtained as candidate inhibitors. When further confirmation of their inhibition activity on cellular KDM5A was made by immunostaining H3K4me3 in KDM5A-overexpressing cells, ryuvidine clearly repressed H3K4me3 demethylation. Ryuvidine prevented generation of gefitinib-tolerant human small-cell lung cancer PC9 cells and also inhibited the growth of the drug-tolerant cells at concentrations that did not affect the growth of parental PC9 cells. Ryuvidine inhibited not only KDM5A but also recombinant KDM5B and C; KDM5B was the most sensitive to the inhibitor. These results warrant that ryuvidine may serve as a lead compound for KDM5 targeted therapeutics.

## Introduction

Epigenetic regulation has been shown to play an important role in gene transcription in eukaryotic cells^1^. Epigenetic regulation involves DNA methylation and histone modifications that occur at multiple sites with different chemical properties. These modifications can affect chromatin structure and thereby gene transcription, and thus govern gene expression^2–4^. DNA methylation and histone modifications are reversible and dynamically controlled depending on the cellular context. Recent research has demonstrated the involvement of epigenetic regulation in critical processes, such as development and disease.

In comparison with simple methylation of cytosine in CpG islands, histone modification is much more complex, not only in the site that is modified, but also chemical properties. Histones H3 and H4 have an amino terminal tail that protrude from the nucleosome, and these tails undergo a variety of covalent modifications such as methylation of lysine and arginine residues, acetylation of lysine and phosphorylation of serine and threonine (see ref.^5^ for nomenclature of chromatin-modifying enzymes).

Methylation of lysine in H3 occurs at various residues, including histone 3 lysine 4 (H3K4), H3K9, H3K27, H3K36 and H3K79, and methylation at different sites gives rise to different effects on gene transcription. For example, methylation of H3K4, H3K36 and H3K79 usually activates transcription, while methylation of H3K9 and H3K79 represses transcription. Furthermore, lysine residues can be mono-, di- or tri-methylated, and different degrees of methylation at the same lysine are thought to exhibit different effects on chromatin structure and transcription^6^.

Several methyltransferases have been proposed as enzymes that methylate H3K4, including SET1, whereas the KDM5 (also known as JARID1) and KDM1 (also known as LSD) families function as demethylases and remove methyl groups with different specificity^6^. KDM1 enzymes demethylate mono-methylated H3K4 (H3K4me1) or di-methylated H3K4 (H3K4me2), while KDM5 enzymes demethylate H3K4me2 or tri-methylated H3K4 (H3K4me3). The KDM5 family contains four members (A, B, C and D), and the KDM1 family contains two members (KDM1A/LSD1 and KDM1B/LSD2). These two families show different cofactor requirements, reflecting their demethylation mechanisms. KDM5 enzymes remove methyl groups via 2-oxoglutarate (2-OG)- and Fe(II)-dependent hydroxylation, and KDM1 enzymes remove methyl groups via flavin-dependent monoamine oxidation.

Numerous studies have shown that KDM5 family members (KDM5A/JARID1A/RBP2^7–14^, KDM5B/JARID1B/PLU-1^15–24^, KDM5C/JARID1C/SMCX^25–30^ and KDM5D/JARID1D/SMCY^30–32^) play a role in oncogenesis. Furthermore, these enzymes have also been shown to mediate drug tolerance in cancer^10,23,33–36^. These findings have encouraged the development of KDM5 inhibitors^37,38^. Indeed, some compounds that show promise as potential drugs have been reported^39–45^.

Here we performed screening of KDM5A inhibitors from small molecule libraries using AlphaScreen technology and identified a battery of screening hits. Further cell-based screening assays narrowed these hits to a panel of potential KDM5A inhibitors. We show that ryuvidine, one of the identified inhibitors in this study, repressed the growth of drug-resistant cells derived from the PC9 non-small-cell lung cancer (NSCLC) cell line.

## Results

### *In vitro* screening of KDM5A inhibitors

A schematic of the screening strategy using AlphaScreen technology (PerkinElmer) that we used to identify KDM5A inhibitors is shown in Supplementary Fig. 1A. KDMA5A-catalyzed demethylation of biotinylated H3K4me3 peptide produces biotinylated H3K4me2 and H3K4me1. These products selectively bind to the acceptor beads coated with antibody against H3K4me2/me1 peptides and also bind to the streptavidin-coated donor beads via biotin in the peptides, producing a ternary complex of peptide-acceptor bead-donor bead. A photosensitizer in the donor bead generates singlet oxygen upon excitation at 680 nm and the singlet oxygen excites a chromophore in the acceptor bead in the ternary complex, emitting light at around 615 nm. In this experiment, we used an antibody that recognizes both H3K4me2 and H3K4me1 peptides.

His-tagged KDM5A1–797 was produced in Sf9 insect cells and affinity purified using the His tag. Kinetic analyses, including the dependency of the reaction on enzyme quantity, reaction time, and concentrations of cofactors (2-OG and Fe(II)) and substrate, confirmed that the purified KDM5A was suitable for screening for inhibitors (Supplementary Fig. 1B–F).

We screened 3,865 small molecules from known drug libraries (Microsource International Drug, MicroSource US Drug, Prestwick, and Tocris, the libraries consisting molecules with known effects on biological processes). We selected 60 compounds that showed inhibition rates higher than 70% at 5 μM. In the standard assay condition, Fe(II) was included at 3 μM. We confirmed that the inhibitory activity of the 60 compounds was not significantly repressed in the presence of 50 μM Fe(II), which excluded the possibility that the compounds exhibited inhibitory effects through chelating Fe(II) or competing with Fe(II).

### Cell-based screening of KDM5A inhibitors

To identify KDM5A inhibitors that function in cells, we developed a reporter assay in which inhibitor-mediated activation of a promoter increased luciferase reporter activity.

We first conducted cDNA microarray analysis on mRNA from PC9 parental cells and KDM5A-overexpressing PC9 cells and found that TFPI-2 (tissue factor pathway inhibitor-2) mRNA was reduced to 19% in KDM5A-overexpressing cells (Supplementary Table 1). We also previously demonstrated that knockdown of LSD1 and LSD2 in HEK293 cells induced TFPI-2 expression^46^. Together these findings suggest that the TFPI-2 promoter may respond to KDM5A inhibitors.

**Figure 1:**
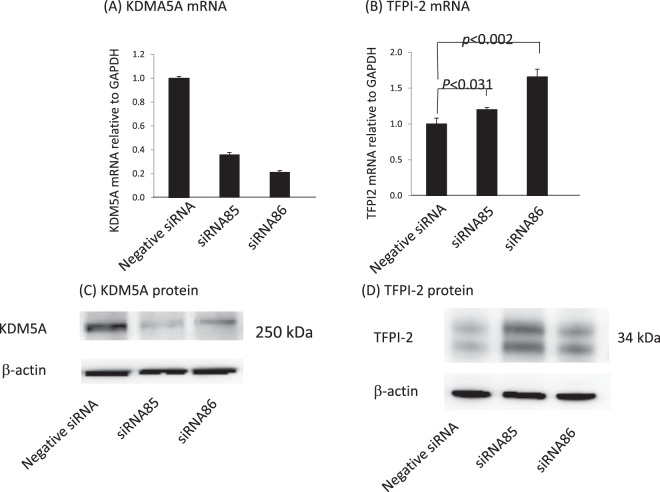
Knockdown of KDM5A in HEK293 cells promotes expression of TFPI-2. RNA and protein were extracted at 48 h after introduction of siRNAs but those of TFPI-2 were extracted at 96 h after siRNA introduction. (**A**) siRNA85 and 86 significantly reduced KDM5A mRNA compared with negative control (NC) siRNA. GAPDH mRNA was used for normalization. (**B**) TFPI-2 mRNA was significantly increased upon KDM5A knockdown. (**C**) KDM5A protein was decreased upon siRNA-mediated knockdown. (**D**) TFPI-2 protein was increased upon KDM5A knockdown by siRNA. Data are shown as means ± SD (n = 3).

To validate this possibility, we performed knockdown of the KDM5A gene in HEK293 cells by transfection with two siRNAs. Both siRNA85 and siRNA86 significantly decreased KDM5A mRNA compared with negative control siRNA (Fig. 1A) with a parallel increase in TFPI-2 mRNA (Fig. 1B). Western blotting confirmed that KDM5A was reduced upon siRNA-mediated knockdown (Fig. 1C), while TFPI-2 was elevated (Fig. 1D). The doublet bands of TFPI-2 reflect differential glycosylation^47^. These results suggest that expression of a TFPI2 promoter-directed reporter luciferase gene may respond to KDM5A inhibitors.

**Figure 2:**
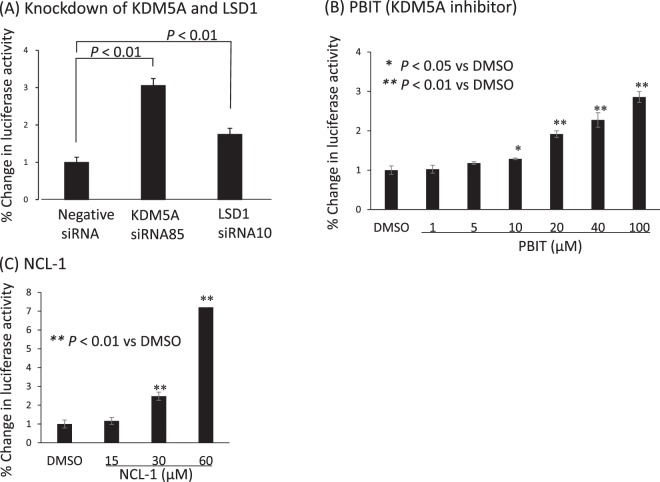
Reporter assay with HEK293TFPI-2-Luc cells. A HEK293 cell line was established that expressed the luciferase gene driven by the TFPI-2 promoter. (**A**) Knockdown of KMD5A and LSD1 increases activity of the luciferase reporter driven by the TFPI-2 promoter. The HEK293 stable cell line was transfected with the indicated siRNAs, and luciferase activity was assayed 72 h later. Luciferase levels in control transfected cells were set as 100%. (**B**) PBIT, a KDM5A inhibitor, increases reporter activity. Luciferase activity was assayed at 48 h after addition of PBIT. (**C**) NCL-1, a LSD1 inhibitor, increases reporter activity. Luciferase activity was assayed at 72 h after addition of NCL-1. Error bars show the means ± SD (n = 3). *

We then constructed a reporter plasmid in which the human TFPI-2 promoter region (−513 to +53) was subcloned upstream of the luciferase gene. A stable HEK293 clone, HEK293TFPI-2-Luc cells that harboured the reporter plasmid was established, and we then examined whether the reporter responded to knockdown of KDM5A. Indeed, knockdown of KDM5A by siRNA significantly elevated the expression of luciferase and, as expected from our previous result^46^, knockdown of LSD1 by siRNA10 also increased reporter expression (Fig. 2A). We next examined whether the reporter responded to a known KDM5A inhibitor (PBIT) that inhibits cellular KDM5A^39^. PBIT induced reporter expression in a dose-dependent manner (Fig. 2B), validating that this reporter assay would enable screening KDM5A inhibitors that function in cells. Notably, an LSD inhibitor (NCL-1)^46,48,49^ also increased the reporter activity (Fig. 2C), indicating this assay is responsive to inhibitors for LSD as well as KDM5A.

The 60 identified compounds were subjected to the reporter assay. We selected 10 compounds that increased luciferase activity by more than two-fold at 10 μM (Table 1). Table 1 also includes their IC_50_ values determined by AlphaScreen technology. A dose-dependent inhibition curve to measure IC_50_ is shown in Supplementary Fig. 2, which shows the inhibition curve of ryuvidine as a representative compound.

**Table 1:** Potential KMD5A inhibitors identified by cell-based reporter assay.

Compound	IC_50_ (μM)^a^
Ryuvidine	1.4
Proflavine Hemisulfate	1.8
NSC95397	0.055
BVT948	2.6
Nitroxoline	5.0
Tolonium Chloride	1.7
Methylene Blue	1.1
Thimerosal	0.13
Pyrithione Zinc	0.32
Auranofin	1.6

^a^IC_50_ values were determined with AlphaScreen technology (see also Supplementary Fig. 2 where dose-dependent inhibition by ryuvidine is shown).

These compounds increased luciferase activity of the cell-based reporter assay by more than two-fold at 10 μM.

### MALDI-TOF/MS assay

As mentioned above, the selected compounds may have inhibition activity on LSD. Recombinant LSD1 and LSD2 were prepared and assayed with each of the compounds (at 20 μM) using the MALDI-TOF/MS method, as described previously^50^. Only thimerosal significantly inhibited LSD activity, and the other 9 compounds showed no effects. Thimerosal, therefore, was not used in the subsequent experiments.

**Figure 3:**
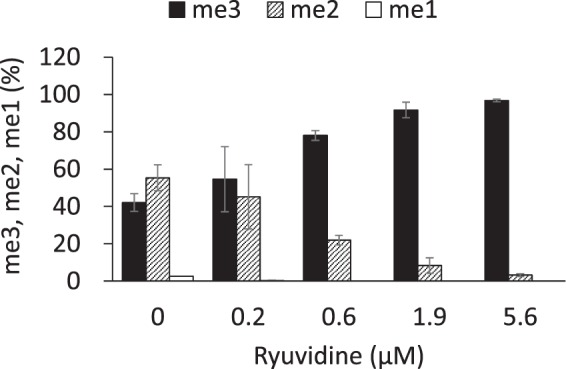
Ryuvidine inhibition of KDM5A was measured by MALDI-TOF/MS methods. Ryuvidine inhibition of KDM5A in the presence of 2-OG was evaluated. Levels of H3K4me3 (me3), H3K4me2 (me2) and H3K4me1 (me1) were measured by MALDI-TOF/MS. H3K4me3 at reaction time 0 min was defined as 100%. Data are presented as means ± SD (n = 3).

To confirm that these 9 compounds inhibited KDM5A, we used the H3K4me3 peptide as substrate and examined enzyme reaction products (H3K4me2/H3K4me1 peptides) using the MALDI-TOF/MS method. The enzyme reaction was dependent on the presence of cofactors (Fe(II) and 2-OG) and reaction time, validating this method for assaying KDM5A (Supplementary Fig. 3). As shown in Fig. 3, ryuvidine repressed a decrease of H3K4me3 and increases of H3K4me2 and H3K4mel in a dose-dependent manner. All of the other 8 compounds also showed similar inhibition (Supplementary Fig. 4). Under the standard reaction conditions, 2-OG was added at 50 μM. When 2-OG was increased to 100 μM, inhibition by ryuvidine was not affected (Supplementary Fig. 5), showing that competition of ryuvidine with 2-OG was unlikely. Similar results were obtained with NSC95397, tolonium chloride, methylene blue, pyrithione zinc and auranofin (Supplementary Fig. 5). However, inhibition by BVT948, proflavine hemisulfate and nitroxoline was significantly reduced by increasing 2-OG (Supplementary Fig. 5), suggesting that inhibition by these three compounds was mediated through competing with 2-OG. Therefore, BVT948, proflavine hemisulfate and nitroxolone were not used for further experiments.

### Immunostaining of HEK293 cells overexpressing Flag-tagged KDM5A

**Figure 4:**
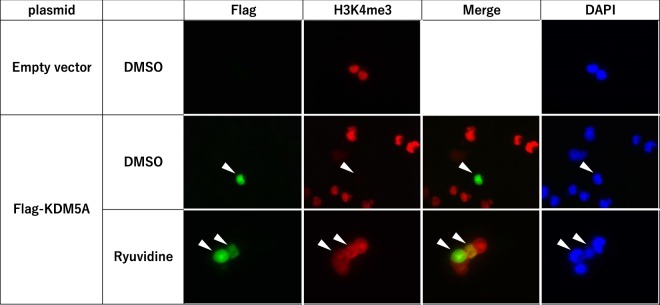
Ryuvidine repressed H3K4 demethylation in HEK293 cells expressing Flag-tagged KDM5A. Flag-tagged KDM5A was transiently overexpressed in HEK293 cells and cells were treated with ryuvidineat 2 uM for 48 h. Flag and H3K4me3 were detected by immunostaining; DAPI staining indicates nuclei. Transfection of empty vector did not produce Flag-positive cells and H3K4me3 was clearly detected. H3K4me3 was markedly reduced in Flag-positive cells in which KDM5A was overexpressed. Ryuvidine repressed the reduction of H3K4me3.

To examine whether the selected inhibitors could prevent reduction of H3K4me3 in cells overexpressing KDM5A, we transiently expressed Flag-tagged KDM5A in HEK293 cells and performed immunostaining with antibodies against Flag and H3K4me3. In Flag-negative cells, H3K4me3 was detected, while H3K4me3 was undetectable in Flag-positive cells (Fig. 4). Notably, when treated with ryuvidine, H3K4me3 was clearly detected in Flag-positive cells. These results suggest that the overexpressed KDM5A demethylated H3K4me3 and ryuvidine prevented the reduction of H3K4me3 through inhibition of KDM5A. However, the other 5 compounds (NSC95397, tolonium chloride, methylene blue, pyrithione zinc and auranofin) did not show clear repression of the reduction of H3K4me3 (Supplementary Fig. 6), although they induced luciferase activity in the reporter assay. This discrepancy between ryuvidine and other compounds remains to be studied.

### Drug-resistant non-small-cell lung cancer PC9 cells

PC9 cells express a mutant form of EGFR (epidermal growth factor receptor) with a 15 bp deletion in exon 9 (E746–A750), resulting in a constitutively active form of EGFR-TK (EGFR-tyrosine kinase)^51^. Gefitinib was developed as an EGFR-TK inhibitor and remarkably reduces tumour growth in many lung cancer patients; however, a drug-resistant cancer cell population emerges after treatment, resulting in recurrence of the disease. Sharma *et al*.^33^ found that most PC9 cells cultured in the presence of high concentration of gefitinib died, but a small population of the cells could survive and proliferate, giving rise to drug-tolerant cells, called DTEPs (drug-tolerant expanded persisters). Further analyses of DTEPs suggested that the elevated expression of KDM5A was attributable to the appearance of these drug-tolerant cells.

We next considered whether the KDM5A inhibitors may repress growth of DTEPs more efficiently than parental PC9 cells. To address this question, we prepared DTEPs according to the reported procedures^33^. When PC9 cells were cultured in the presence of high concentration of gefitinib (2 μM) for 3 days, most of the cells died, but a small population of cells survived and continued growing (Supplementary Fig. 7A). We continued to culture these cells for 2 months with 2 μM gefitinib, and the resulting cells were used as DTEPs. Small but reproducible increases of KDM5A mRNA and demethylase activity were observed in DTEPs compared with parental PC9 cells (Supplementary Fig. 7B and C). DTEPs were resistant to various concentrations of gefitinib, while parental PC9 cells quickly died under these conditions (Supplementary Fig. 7D). In addition to deletion of exon 9, other EGFR mutations (L858R and T790M) giving rise to gefitinib-resistant cells were identified in NSCLC^52,53^. We confirmed that the exon 9 deletion in EGFR was present in both PC9 cells and DTEPs and that both cell lines were absence for the L858R and T790M mutations.

**Figure 5:**
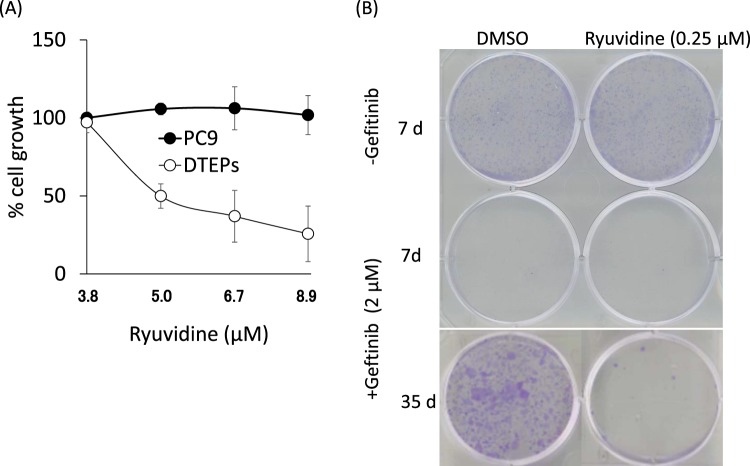
Effect of ryuvidine on gefitinib-tolerant PC9 cells (DTEPs). (**A**) Ryuvidine inhibits growth of gefitinib-tolerant PC9 cells (DTEPs) but not parental PC9 cells. MTT values in the presence of DMSO alone were defined as 100%. Error bars show the means ± SD (n = 3). (**B**) Ryuvidine represses generation of DTEPs.

We next examined the effects of KDM5A inhibitors on the growth of PC9 cells and DTEPs. Remarkably, ryuvidine inhibited the growth of DTEPs at concentrations in which PC9 cells were mostly unaffected (Fig. 5A). Other 5 compounds, however, did not show marked differential effects on PC9 and DTEPs (Supplementary Fig. 8). Furthermore, co-treatment of gefitinib and ryuvidine in PC9 cultures impeded generation of DTEPs colonies (Fig. 5B).

### Specificity of ryuvidine inhibition

We next examined the specificity of ryuvidine in inhibiting other KDM5 family members. Ryuvidine inhibited KDM5A, B, and C with different efficiencies (Table 2). KDM5B was the most sensitive to ryuvidine, whereas KDM5C showed more resistance to the drug. LSD activities were not inhibited at a high concentration of ryuvidine (50 μM).

**Table 2:** Specificity of ryuvidine.

Enzymes	IC_50_
KDM5A	0.57 ± 0.1 μM^a^
KDM5B	0.026 ± 0.01 μM^a^
KDM5C	8.73 ± 1.8 μM^a^
LSD1	no inhibition at 50 μM^b^
LSD2	no inhibition at 50 μM^b^

Activities were measured with MALDI-TOF MS method. Ryuvidine concentration was varied from 0.21–50 µM for KDM5A and KDM5C and 0.008–50 µM for KDMA5B.

^a^Data represent n = 3; ^b^Data represent n = 9.

## Discussion

To identify small molecules that inhibit KDM5A, we used AlphaScreen technology to screen drug libraries consisting of 3,865 compounds with known effects on cells and obtained 60 compounds that were positive in the *in vitro* assay. These compounds were subjected to further evaluation using a reporter assay to determine their ability to inhibit cellular KDM5A. The reporter assay was based on our finding that siRNA-mediated knockdown of KDM5A in HEK293 cells increased TFPI-2 expression. We prepared an HEK293 clone that harboured a reporter plasmid in which luciferase expression was under control of the TFPI-2 promoter region (−513 to +53). H3K4me3 is generally increased in the promoters of transcribed genes and thought to be a marker for activated transcription, but only in a subset of genes^38^. We have not yet determined the precise sequence(s) in the TFPI-2 promoter that are responsible for KDM5A knockdown-mediated activation of reporter expression.

We selected the 10 compounds that significantly induced the activity of our reporter by more than two-fold in the luciferase reporter assay. Four compounds (thimerosal, BVT948, proflavine hemisulfate and nitroxoline) were excluded, because thimersal strongly inhibited LSDs and the other three compounds inhibited KDM5A activity through competing with 2-OG, a cofactor of KDM5A. The chemical structures of the final 6 compounds are shown in Supplementary Fig. 9. We examined whether these compounds acted on cellular KDM5A using immunostaining in cells overexpressing KDM5A, and found that only ryuvidine clearly repressed the reduction of H3K4me3(see Fig. 4).

NSCLC-derived PC9 cells are sensitive to the EGFR-TK inhibitor gefitinib, and extended culture of PC9 in the presence of high concentration of the drug produces drug-resistant cells, DTEPs. Induction of KDM5A during culture is responsible for occurrence of the drug tolerance^33^. We confirmed these observations (see Supplementary Fig. 7) and examined the effects of the selected compounds on the growth of DTEPs. Interestingly, ryuvidine inhibited growth of DTEPs at concentrations where growth of parental PC9 was unaffected. Other 5 inhibitors did not show differential effects on both cell types, suggesting that they are toxic. Previous studies have reported that ryuvidine exhibits cytotoxicity by inhibiting CDK4^54^, CDC7 kinase^55^ and SETD8/KMT5A (H3K20 monomethyltransferase)^56^. As these inhibitions are not observed in parental PC9 cells, the contribution of these events to ryuvidine-derived growth inhibition of DTEPs is unlikely. However, the possibility has not been excluded that some of these enzymes in DTEPs have become more sensitive to ryuvidine inhibition than in PC9 cells.

Drug-tolerant cells may be caused by mutations that render key proteins insensitive to drugs and/or activation of an alternative pathway for cell growth. We confirmed that the DTEPs used in this study did not harbour previously identified mutations (L858R and T790M) that make EGFR insensitive to gefitinib. Some studies have reported activation of alternative pathways in gefitinib-tolerant PC9 cells, such as the IGF-1 receptor pathway^33,57^, Akt-β-catenin pathway^58^ and FGF2-FGF receptor 1 autocrine pathway^59^, suggesting diverse drug-resistance mechanisms in PC9 cells^60^. We have not yet explored the molecular mechanism(s) by which our DTEPs emerged.

Although ryuvidine is a compound with known effects on cells, our findings indicate that ryuvidine may be a useful lead chemical for the generation of a new class of KDM5 inhibitors. Chemical modifications of ryuvidine to improve the specificity and efficacy as an H3K4me3me2 demethylase inhibitor are warranted. Poor permeation into cells may be a widespread mechanism that underlies modest performance of a drug. We have shown that conjugation of a proteasome inhibitor (ridaifen-F) to a cell-penetrating peptide markedly elevates the inhibition of cellular proteasome through accelerated uptake^61^, suggesting modification point of ryuvidine. Intracellular modifications would also impair drug efficacy. Ryuvidine, a derivative of benzothiazoledione, may be reduced by the cellular dione system, converting into a compound with low efficacy. This suggests another point for modification of ryuvidine.

Our results showed that NSC95397 inhibited KDM5A at much lower concentrations compared with the other identified compounds (see Table 1). However, this compound did not induce luciferase activity in the reporter assay, suggesting that it might be difficult for NSC95397 to penetrate into cells. Chemical modifications of this compound including conjugation with a cell-penetration peptide might dramatically improve its inhibitory potency of cellular KDM5A.

## Materials and Methods

### Compounds tested as histone demethylase inhibitors

The KDM5A inhibitor PBIT (2–4(4-methlyphenyl)-1,2-benzisothiasol-3(2 H)-one) was purchased from Sigma-Aldrich and a LSD inhibitor, NCL-1 (*N*-((1 S)-3-(3-(*trans*-aminocyclopropyl)phenoxy-1-(benzylcarbamoyl)propyl)benzamide)^49^ was kindly provided by Prof. Takayoshi Suzuki of Kyoto Prefectural University of Medicine. Compounds were dissolved in 5% DMSO.

### Cell culture

HEK293 cells were obtained from the RIKEN Cell Bank (Ibaraki, Japan; Cell No. RCB1637) and maintained in Dulbecco’s modified Eagle’s Medium (DMEM) (D5796; Sigma, St. Louis, MO, USA) supplemented with 10% (v/v) fetal bovine serum (FBS) (Nichirei, Tokyo, Japan), 100 μg/mL streptomycin, and 100 U/mL penicillin at 37 °C in a humidified 5% CO_2_ incubator. PC9 cells and PC9-derived DTEPs were cultured in RPMI1640 medium containing the supplements described above.

### Production of His-tagged KDM5 proteins in Sf9 insect cells and purification

Sf-9 cells were infected with baculovirus expressing N-terminally hexahistidine-tagged and C-terminal truncated human KDM5 proteins (KDM5A 1–797, KDM5B 1–769 and KDM5C 1–839). After culture for three days at 28 °C, cells were harvested and lysed. Lysate was cleared by centrifugation, and the supernatant was applied to a HisTrap HP column (0.7 × 2.5 cm) (GE Healthcare) equilibrated with buffer A (20 mM sodium phosphate buffer, pH 7.5, containing 0.5 M NaCl, 40 mM imidazole, 10 mM 2-mercaptoethanol). Elution was performed by linearly increasing the concentration of imidazole from 40 to 500 mM in buffer A. Fractions enriched in enzyme activity were collected and concentrated.

### AlphaScreen method for KDM5A assay

The assay buffer consisted of 50 mM HEPES-KOH pH 7.5, 0.1% BSA, and 0.01% Tween 20. A 0.1-μl sample of compound solution dissolved in DMSO or DMSO alone was spotted into the wells of an AlphaPlate-384 SW (6008350, PerkinElmer) using the EDR-384 UX multi-channel pipettor (BioTec). Assay buffer (5 μl) containing 10 nM His-tagged KDM5A was dispensed into the wells using the mini-Gene LD-01 dispenser (BioTec). The plates were incubated for approximately 20 min at room temperature. Reactions were initiated by the addition of 5 μl of assay buffer containing 60 nM biotinylated H3K4me3 peptide, 80 μM 2-OG, 200 μM ascorbate, and 6 μM (NH4)_2_Fe(SO_4_)·6H_2_O. The final concentrations of each compound from the known drug libraries were 5 μM in the reaction mixture, respectively. As background, 2-OG was omitted from the reaction mixture. The plates were incubated at 26 °C for 1 h and then 8 μl of 1X AlphaLISA Epigenetics Buffer 1 (AL008F, PerkinElmer), containing 22.5 μg/ml anti-H3K4me1-2 AlphaLISA acceptor beads (AL116R, PerkinElmer), 22.5 μg/ml streptavidin-coated donor beads (6760002B, PerkinElmer), and 16.9 mM EDTA (Alpha beads mixture) were added. The plate was incubated at 23 °C overnight. Luminescence was measured using the EnSpire Alpha plate reader (PerkinElmer).

To measure background values, the reactions were run without 2-OG. The control values measured by running the reactions in the absence of compounds were defined as 100% activity. The sample values were measured by running the reactions with a compound for screening. In all mixtures, DMSO was added at final 1%. Inhibition (%) by a compound was calculated as 100 × [1 − (sample-background)/(control-background)].

Prior to experiments, the assay was optimized for reaction time and concentrations of the reaction components (Supplementary Fig. 1B–F).

### Luciferase reporter assay

Genomic DNA was isolated from HeLa cells using the Gentra Puregene Cell Kit. The human TFPI-2 gene 5′ flanking region from −513 to +53 (where +1 is the first transcription start site) was amplified by PCR using HeLa genomic DNA as a template with the forward primer 5′-AATACTCGAGCTGTCCACCTACGATATATTATAAGCCTGT-3′ and reverse primer 5′-TATGAAGCTTGCTGGGCAAGGCGTCCGAGAAAGC-3′. The underlined text indicates restriction enzyme sites for *Xho* I and *Hin*d III, respectively. The amplified fragment was subcloned into the *Xho* I and *Hin*d III sites of the luciferase reporter plasmid pGL4.17 to generate the TFPI2-Luc plasmid.

HEK293 cells were transfected with the TFPI2-Luc plasmid using Lipofectamine 2000 reagent. G418-resistant colonies were cloned and propagated in selective medium containing 0.6 mg/ml G418. One of the stable clones that stably expressed the TFPI2-Luc plasmid was used and referred to as HEK293TFPI2-Luc cells.

HEK293TFPI2-Luc cells were seeded at 3 × 10^4^ cells per well in a 96-well plate in 0.1 ml of DMEM containing 10% FBS. After 24 h, the cells were treated as indicated. Cells were washed with PBS and lysed with 20 μl of Passive Lysis Buffer. Lysates were mixed with 100 μl of luciferase assay reagent in a 384-well plate to assay luciferase activity using a Fusion microplate analyser.

### siRNA transfection

The siRNA duplex oligonucleotides used for knockdown of KDM5A and LSD1 were as follows: KDM5AsiRNA85, 5′-GCACAAGGAUGAACAUUCUTT-3′; JARID1AsiRNA86, 5′-AGAAUGUUCAUCCUUGUGCTT-3′; and LSD1siRNA10, 5′-CACAGGGATCTGACCGCCCTA-3′^23^. AllStars Negative control siRNA (5′-AATTCTCCGAACGTGTCACGT-3′) was obtained from Qiagen K.K.

HEK293 cells were seeded at 1 × 10^5^ cells/well (total volume: 1 mL) in 12-well plates for RNA extraction and 2.5 × 10^5^ cells/well (total volume: 2.5 mL) in 6-well plates for protein extraction. After 24 h, Lipofectamine RNAiMAX transfection reagent (Invitrogen Inc.) was used to transfect siRNA duplexes (final concentration of 10 nM) according to the manufacturer’s instructions.

### RNA extraction and quantitative reverse transcription-PCR (qRT-PCR)

RNA extraction and qRT-PCR were done as described previously^46^. Primers are as follows: KDM5A FWD, 5′-ATGTGCCCAAAGGAGACTGG-3′; KDM5A REV, 5′- GCCGCCAAAATTCCTTTTC-3′, LSD1 FWD, 5′-CAGGCTTGGCAGCAGCTCGA-3′; LSD1 REV, 5′-TCTCCACTTCCTGAACGCATC-3′; TFPI-2 FWD, 5′-GTCGATTCTGCTGCTTTTCC-3′; TFPI-2 REV, 5′-CAGCTCTGCGTGTACCTGTC-3′; GAPDH FWD, 5′-CAGCCTCAAGATCATCAGCA-3′; and GAPDH REV, 5′-TGTGGTCATGAGTCCTTCCA-3′.

### Western blotting

Western blotting of HEK293 lysates were done as described previously^46^. Antibodies were rabbit anti-KDM5A (NB110-40499; Novus Biologicals Inc., Centennial, USA; 1:2,000 dilution), anti-LSD1 (ab17721; Abcam Inc., Cambridge, UK; 1:2,000 dilution), anti-Flag (M185; MBL Inc., Nagoya, Japan; 1:2,000 dilution), anti-TFPI2 (sc-48380; Santa Cruz Biotechnology Inc., Santa Cruz, CA; 1:1,000 dilution), or anti-β-actin (sc-47778; Santa Cruz Biotechnology Inc.; 1:1,000 dilution).

### Matrix-assisted laser desorption ionization time-of-light mass spectrophotometry (MALDI-TOF MS)

Histone demethylation reactions were conducted at 37 °C in 20 μl of 50 mM HEPES-KOH (pH 7.5) containing 15 μM H3K4me3 peptide, 10 μM Fe(II), 100 μM ascorbate, 50 μM 2-OG, and 3 μg KDM5A, B or C. H3K4me3 substrate and demethylated products (H3K4me2 and H3K4me1) were measured with MALDI-TOF MS, as described previously^49^.

### Preparation of LSD1 and LSD2 and MALDI-TOF MS

Production and purification of His-tagged human full-length LSD1 and His-taggedΔN25 human LSD2 were performed as described previously^23^. Histone demethylation was performed at 37 °C for 30 min in 20 μl of 50 mM HEPES-NaOH (pH 7.5 for LSD1 or pH 8.5 for LSD2) containing 0.1% BSA, H3K4me2 peptide (30 μM for LSD1 or 10 μM for LSD2), and 0.25 μg LSD1 or 2 μg LSD2. H3K4me2 substrate and demethylated products (H3K4me1 and H3K4me0) were measured with MALDI-TOF MS as described previously^49^.

### Construction of Flag-KDM5A plasmid

The N-terminally Flag-tagged *KDM5A* gene was PCR amplified from HeLa cells with the forward primer 5′- AAGGTACCATGGACTACAAAGACGATGACGACAAGATGGCGGGCGTGGGGCCG-3′ and reverse primer 5′- TCTAGAGTCGCGGCCGCCTAACTGGTCTCTTTAAGATCCTCCATTGG-3′. The underlined text indicates restriction enzyme sites for *Kpn* I and *Not* I, respectively. The amplified DNA was subcloned into pEGFP-N1 (Clontech) to replace the EGFP gene at the KpnI and Not I sites, generating the Flag-KDM5A plasmid.

### Immunostaining

HEK293 cells (2 × 10^5^ cells/well) were plated on glass coverslips in 12-well plates and cultured for 2 days. Cells were transfected with Flag-KDM5A plasmid or empty vector using FuGENE HD Transfection Reagent (Promega). After 24 h, culture media were changed and inhibitors (2 μM) or DMSO (0.1%) were added. After 2 days, coverslips were washed with PBS and the cells were fixed in 70% methanol, followed by blocking for 30 min with 20% (v/v) Blocking One (Nacalai)/Milli-Q water. Cells were then incubated for 60 min with primary antibodies (anti-Flag antibody (M185-3S; MBL Inc.; 1:3,000 dilution) and anti-H3K4me3 antibody (39915; Active Motif Inc.; 1:1500 dilution). The cells were washed with PBS and incubated for 60 min with secondary antibodies as follows: anti-mouse IgG conjugated Alexa488 (A11029; Cell Signaling Technology Inc.; 1:1,000 dilution) to detect Flag and anti-rabbit IgG conjugated CF555 (20033; Biotium Inc.; 1:1,000 dilution) to detect H3K4me3. After washing with PBS, the cells were stained with DAPI and mounted for viewing.

### Cell proliferation assay

Cells were seeded in a 96-well tissue culture plate at 1 × 10^4^–5 × 10^4^ cells/ml and cultured. Next, 10 μl of 5 mg/ml MTT (Sigma-Aldrich) were added to each well and cells were incubated for 3 h. The medium was discarded and formazan was dissolved in 100 μl lysis buffer (0.4 M HCl in 2-propanol). Optical densities were measured at 570 nm and background was subtracted at 750 nm.

### Gefitinib-tolerant PC9 cells (DTEPs)

DTEPs were prepared as described in Supplementary Fig. 7. PC9 cells and DTEPs were plated at 5000 cells/well in 96 well plates. DTEPs were cultured with 2 μM gefitinib. After 1 day, ryuvidine at varying concentrations or DMSO at 0.24% as controls was added. At 48 h after culture, cell growth was evaluated with MTT assay. When it was examined whether or not ryuvidine prevented generation of DTEPs, PC9 cells were plated (2000 cells/well in 6 well plates) and cultured for indicated periods with or without 2 μM gefitinib. Cells were also cultured in the presence of 0. 25 μM ryuvidine or 0.1% DMSO. Medium was changed at every 3 days. Cells were stained with crystal violet and imaged.

### Statistical analysis

Data were analysed by the Student’s t-test with Welch’s correction. *p* < 0.05 was considered statistically significant.

## Supplementary information

Supplementary Table and Figures and Legends

## Data Availability

All data generated or analysed during this study are included in this published article and its Supplementary Information files.
