# Supplementary material for: Identification of ryuvidine as a KDM5A inhibitor
Source: Sci Rep. 2019 Jul 9;9:9952. doi: 10.1038/s41598-019-46346-x (PMC6616564; doi:10.1038/s41598-019-46346-x)
Supplement: Supplementary file 1 — Supplementary Table and Figures and Legends [file 41598_2019_46346_MOESM1_ESM.pdf]

Supplementary Information

Title

Identification of ryuvidine as a KDM5A inhibitor

Eishin Mitsui[1], Shogo Yoshida[1], Yui Shinoda[1], Yasumasa Matsumori[1], Hiroshi Tsujii[1], Mie Tsuchida[1], Shuichi Wada[1], Makoto Hasegawa[1], Akihiro Ito[2, 3], Koshiki Mino[1, 4], Tetsuo Onuki[4], Minoru Yoshida[3, 4, 5], Ryuzo Sasaki[1, 6], Tamio Mizukami[1, 6*]

[1]Nagahama Inst. Bio-Sci. & Tech., 1266 Tamura, Nagahama, Shiga 526-0829 Japan. [2]Sch. of Life Sci., Tokyo Univ. of Pharm. & Life Sci., 1432-1 Horinouchi, Hachioji, Tokyo 192-0392 Japan. [3]Chem. Genomics, RIKEN CSRS, 2-1 Hirosawa, Wako, Saitama 351-0198, Japan. [4]Drug Discov. Seed Cpds. Explorat. Unit, RIKEN CSRS, 2-1 Hirosawa, Wako, Saitama 351-0198, Japan. [5]Dept. of Biotechnol., The Univ. of Tokyo, 1-1-1 Yayoi, Bunkyo-ku, Tokyo 113-8657 Japan. [6]Frontier Pharma Inc., 1281-8 Tamura, Nagahama, Shiga, 526-0829 Japan.

[*]Correspondence and requests for materials should be addressed to T.M. (email: mizukami@nagahama-i-bio.ac.jp)

Supplementary Figure Legends

Supplementary Fig. 1 AlphaScreen assay of KDM5A. (A) Assay method of KDM5A inhibitors. (B) Time course with different enzyme amounts. Data show average values (n=2). (C) Optimization of enzyme concentrations. Reactions were carried out for 60 min with or without 2-OG. Data show average values (n=2). (D) Optimization of substrate concentrations. Reactions were carried out for 60 min with or without 2-OG. Data are shown as means ± SD (n=3). (E) Optimization of 2-OG concentrations. Reactions were carried out for 60 min. Data are shown as means ± SD (n=3). (F) Optimization of Fe(II) concentrations. Reactions were carried out for 60 min with or without 2-OG. Data are shown as means ± SD (n=3).

Supplementary Fig. 2 Dose-dependent inhibition of KDM5A by ryuvidine. Activity was measured with AlphaScreen assay. Reactions were carried out for 60 min. Activity at DMSO was defined as 100%. Data show average values (n=2).

Supplementary Fig. 3 KDM5A assay with MALDI-TOF/MS method. (A) Mass spectrum of H3K4me3 (me3), H3K4me2 (me2) and H3K4me1 (me1). The reaction was carried out for 10 min. H3K4me3 at time 0 was defined as 100%. (B) Time course. H3K4me3 at reaction time 0 was defined as 100%. Data are shown as means ± SD (n=3). When reactions were carried out without 2-OG or Fe(II) for 30 min, H3K4me3 did not decrease and H3K4me2 and H3K4me1 were not produced.

Supplementary Fig. 4 Inhibition of KDM5A was assayed with MALDI-TOF/MS method. KDM5A reactions were carried out for 10 min in the presence of inhibitors at indicated concentrations. Other conditions were described in Materials and Methods . H3K4me3 at time 0 was defined as 100%. me3, me2 and me1 indicate H3K4me3, H3K4me2, and H3K4me1, respectively.

Supplementary Fig. 5 Effects of 2-OG concentrations on inhibitors of KDMA5A. H3K4me3 was measured by MALDI-TOF/MS method. The reactions were carried out for 30 min with compounds at the indicated concentrations in the presence of 50 or 100 mM 2-OG. H3K4me3 at reaction time 0 was defined as 100%. Data are shown as means ± SD (n=3). $p > 0.05$ was considered not significant statistically (ns)..

Supplementary Fig. 6 Effect of KDM5A inhibitors on H3K4 demethylation in HEK293 cells expressing Flag-tagged KDM5A. Flag-tagged KDM5A was transiently overexpressed in HEK293 cells and cells were treated with the indicated KDM5 inhibitors (NSC95397, tolonium chloride, methylene blue, pyrithione zinc and auranofin) at 2 uM for 48 h. Flag and H3K4me3 were detected by immunostaining; DAPI staining indicates nuclei.

Supplementary Fig. 7 Comparison of properties of PC9 parental cells and gefitinib-tolerant PC9 cells (DTEPs). (A) Generation of gefitinib-tolerant PC9 cells. PC9 cells were plated in 10 cm dishes (10^5 cells/dish) and after 1 day of culture, gefitinib (2 uM) was added. Medium was changed every 3 days. The cells were mostly dead at day 3, but gefitinib-tolerant colonies appeared at day 6 and continued to grow. DTEPs were obtained after culture for 56 days and used for experiments (B, C and D). (B) KDM5A mRNA was increased in DTEPs. Three separate RNA preparations from PC9 and DTEPs were prepared and KDM5A mRNA was quantified. Data are shown as means ± SD (n=3). (C) H3Km4me3 level was decreased in DTEPs. (D) Growth of DTEPs was not repressed by high concentrations of gefitinib, while growth of PC9 cells was severely inhibited. Both cell lines were cultured in the presence of gefitinib at indicated concentrations for 2 days, and cell growth was evaluated with MTT assay. Data are shown as means ± SD (n=3).

Supplementary Fig. 8 Effect of KDM5A inhibitors on growth of PC9 cells and gefitinib-tolerant PC9 cells (DTEPs). Experiments were carried out as described in the legend for Fig. 5. Cells were cultured in the presence of inhibitors at the indicated concentrations and cell growth was measured by MTT assays. Data are shown as means ± SD (n=3).

Supplementary Fig. 9 Structures of inhibitor compounds. Structure of the six inhibitors identified in this study. The compounds inhibit KDM5A *in vitro* (AlphaScreen method and MALDI-TOF/MS assay) and *in vivo* (reporter assay). These compounds do not inhibit LSD. The inhibition of KDM5A is not affected by cofactor concentrations (Fe(II) and 2-OG).

Supplementary Table 1

Gene name [accession]	PC9 signal	PC9/KDM5A signal	Relative expression
GAPDH [NM_002046]	326716	298436	0.91
TFPI-2 [NM_006528]	17858	3349	0.19

Supplementary Fig. 1

(A)

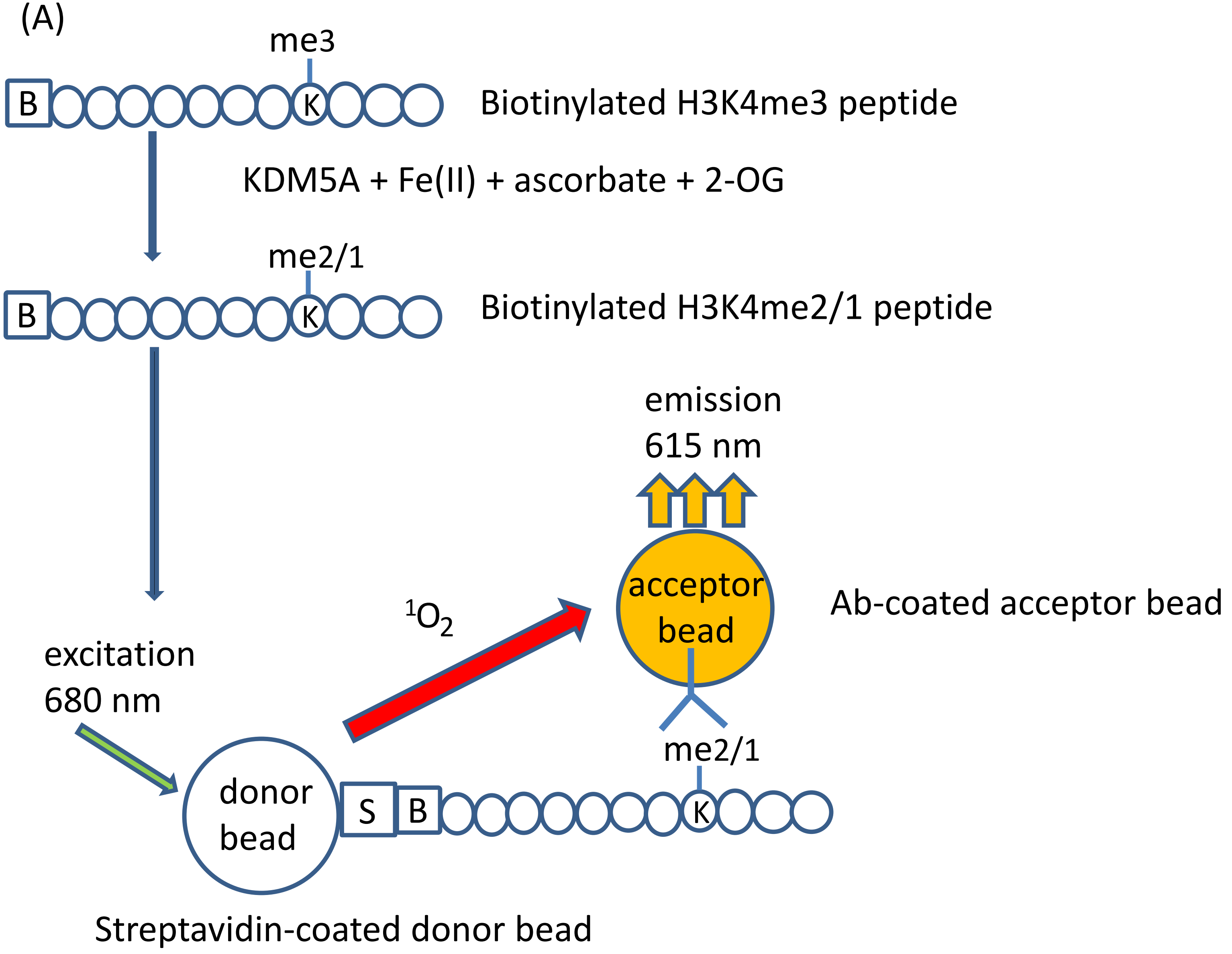

Supplementary Fig. 1

(B)

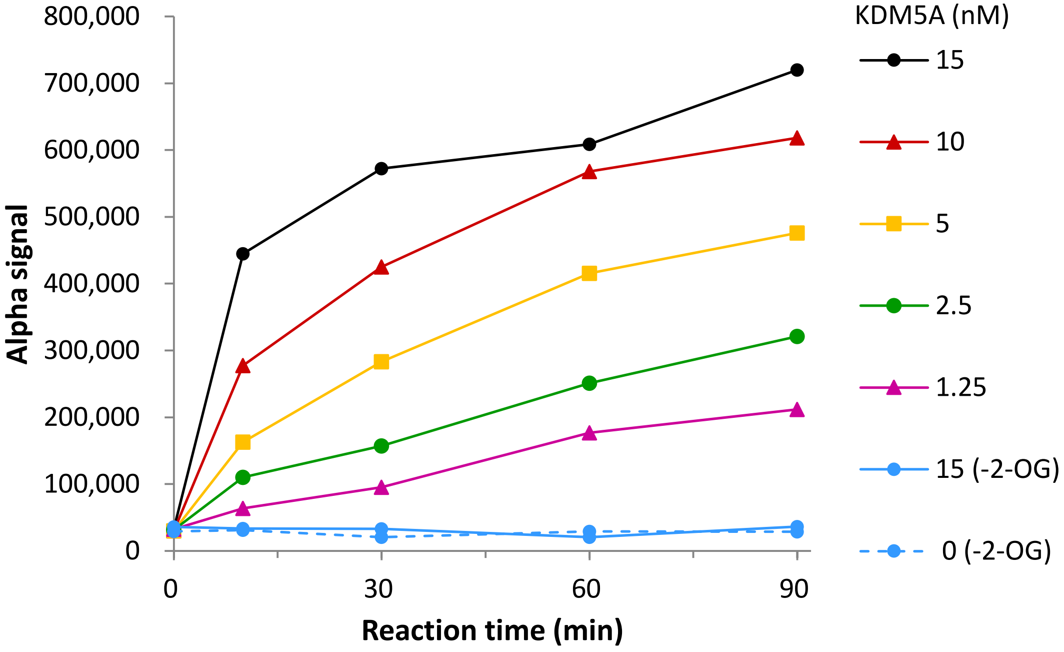

Supplementary Fig. 1

(C)

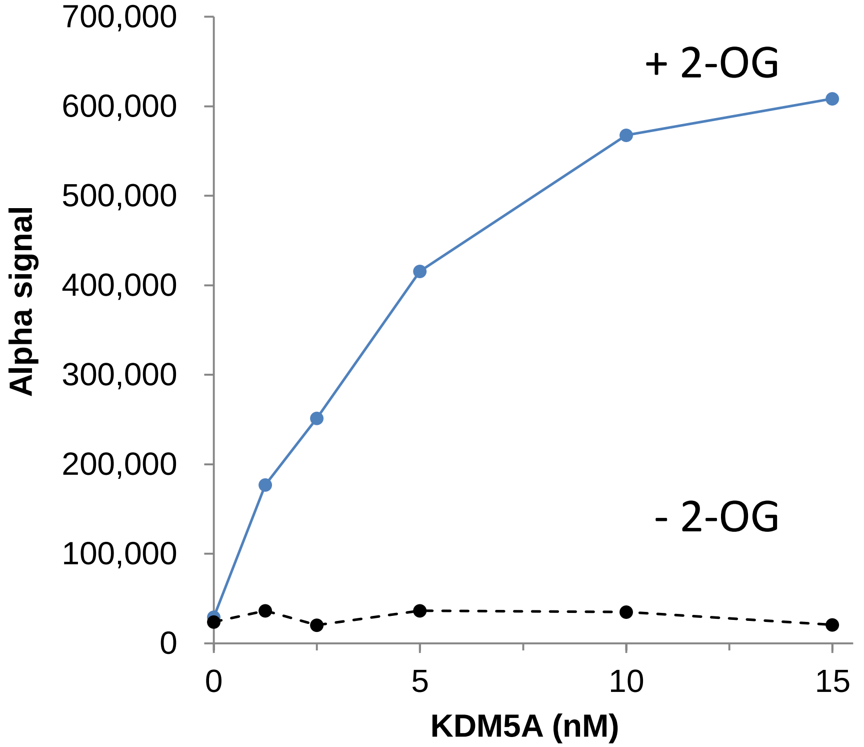

Supplementary Fig. 1

(D)

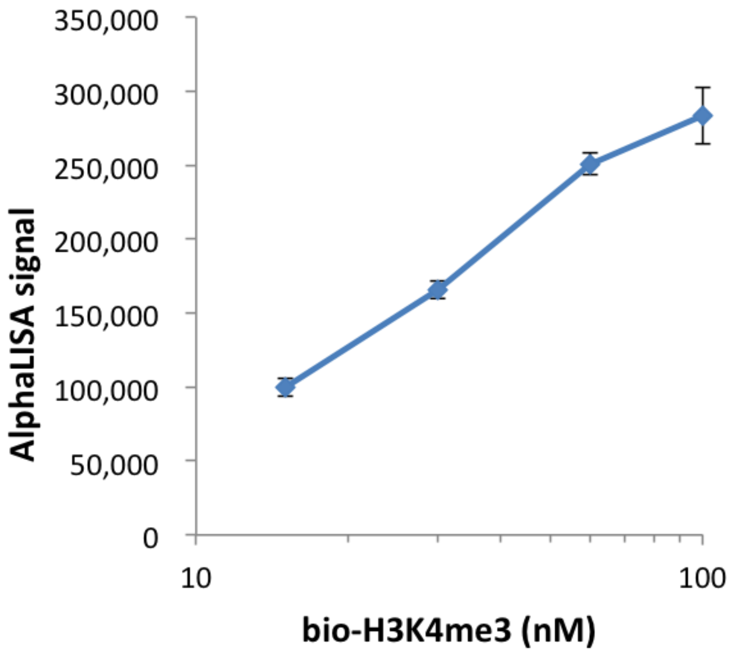

Supplementary Fig. 1

(E)

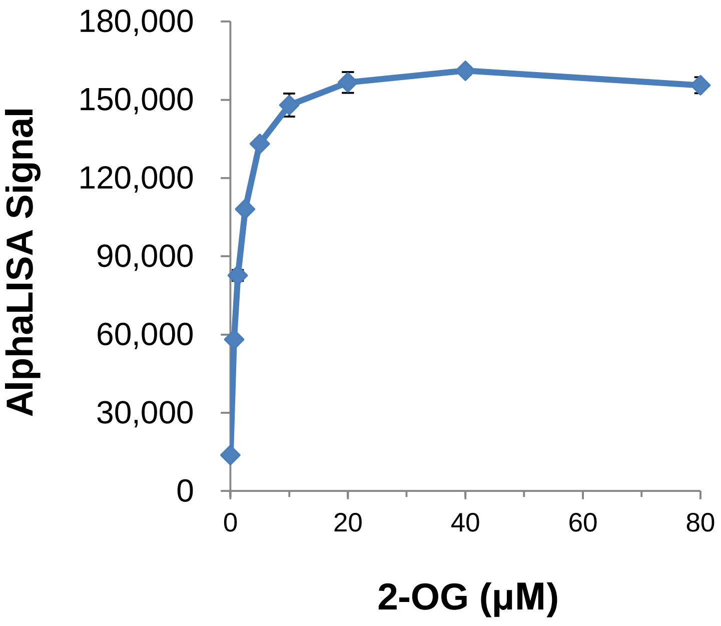

(F)

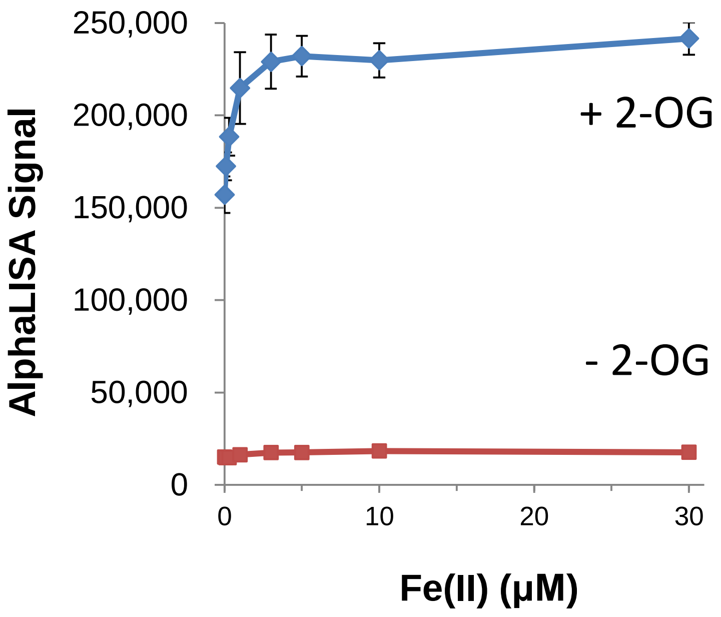

Supplementary Fig. 2

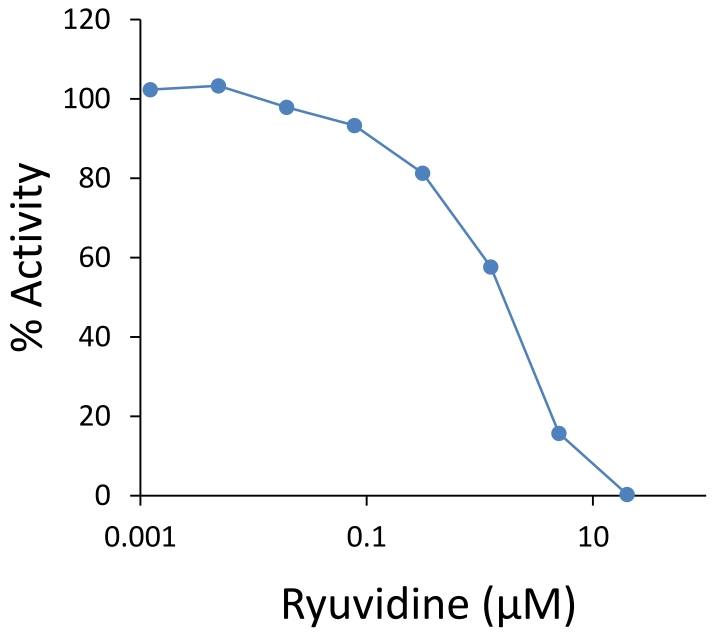

Supplementary Fig. 3

(A)

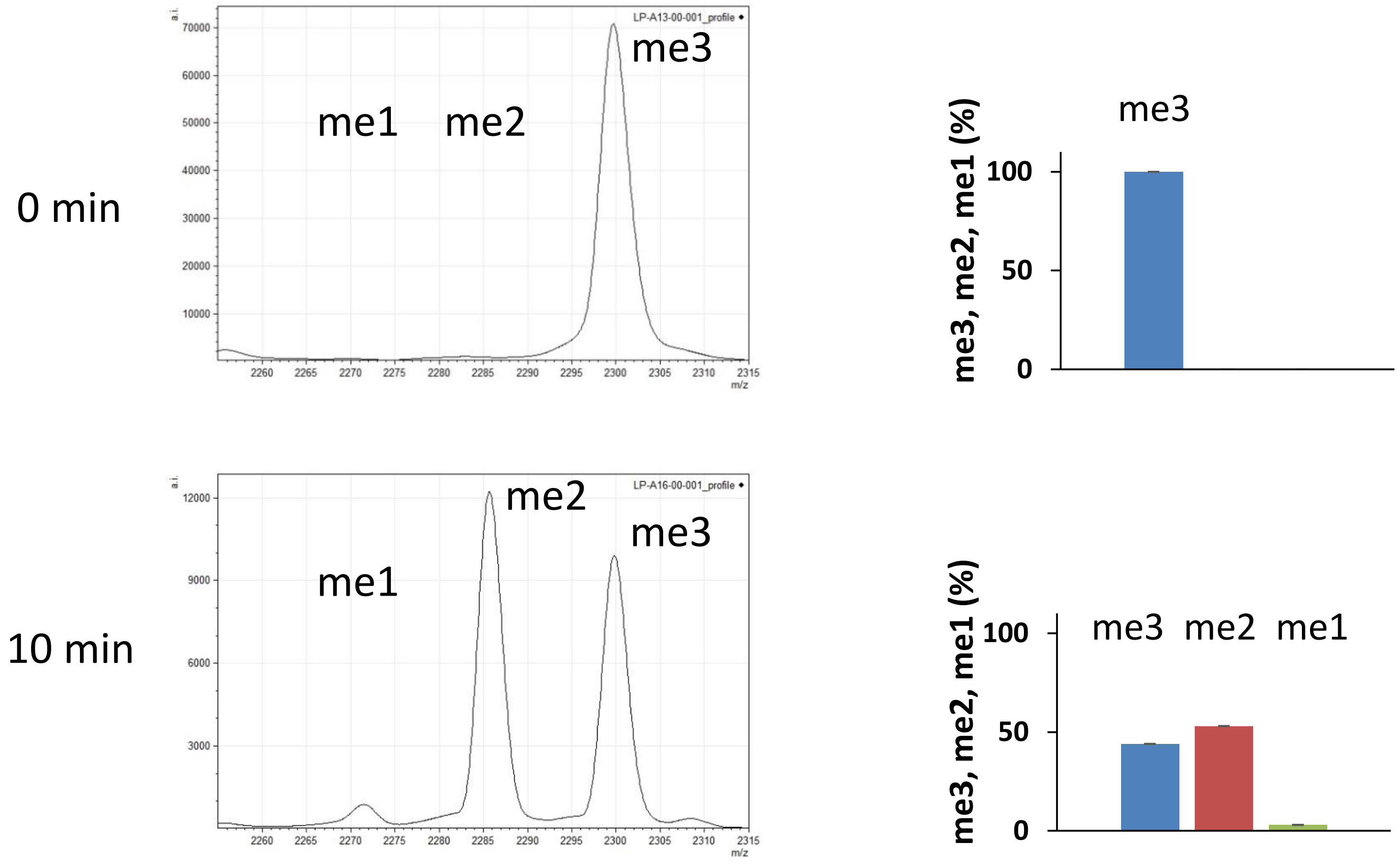

Supplementary Fig. 3

(B)

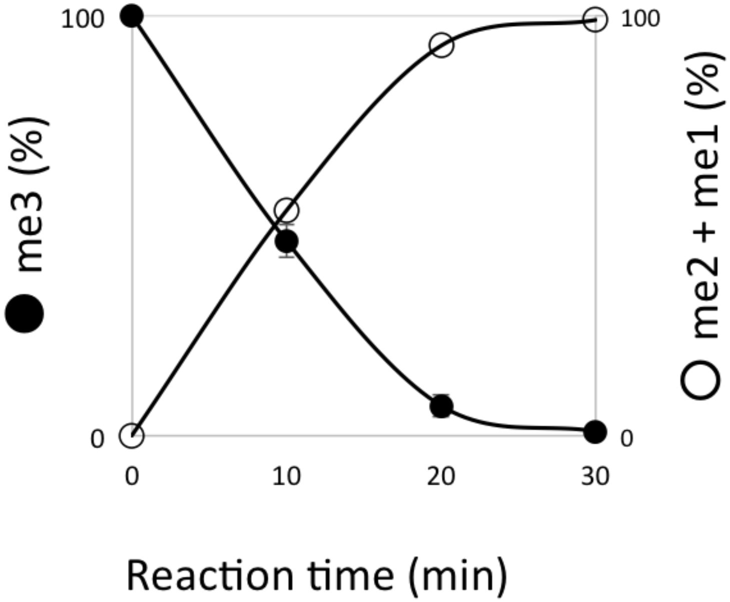

Supplementary Fig. 4

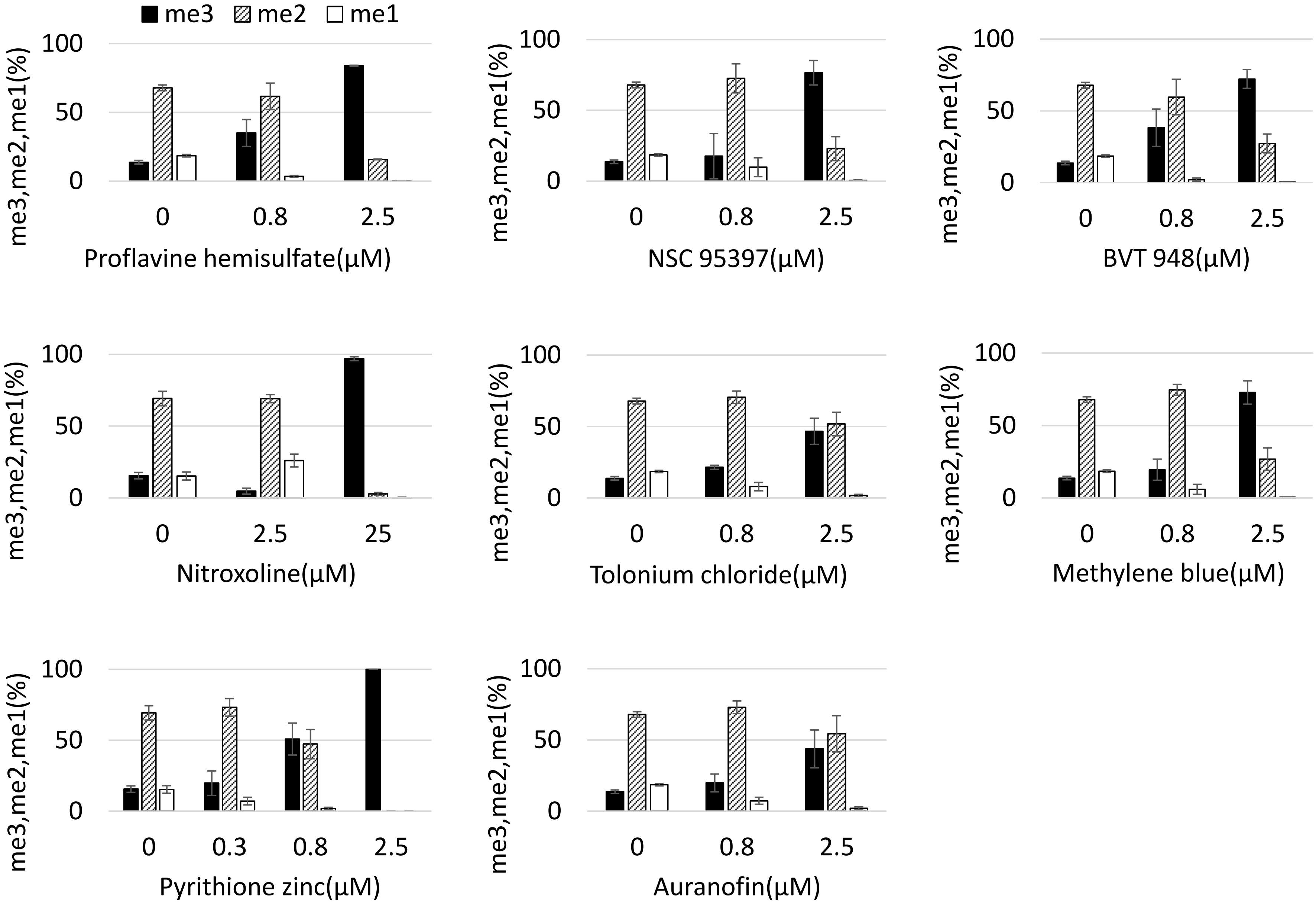

Supplementary Fig. 5

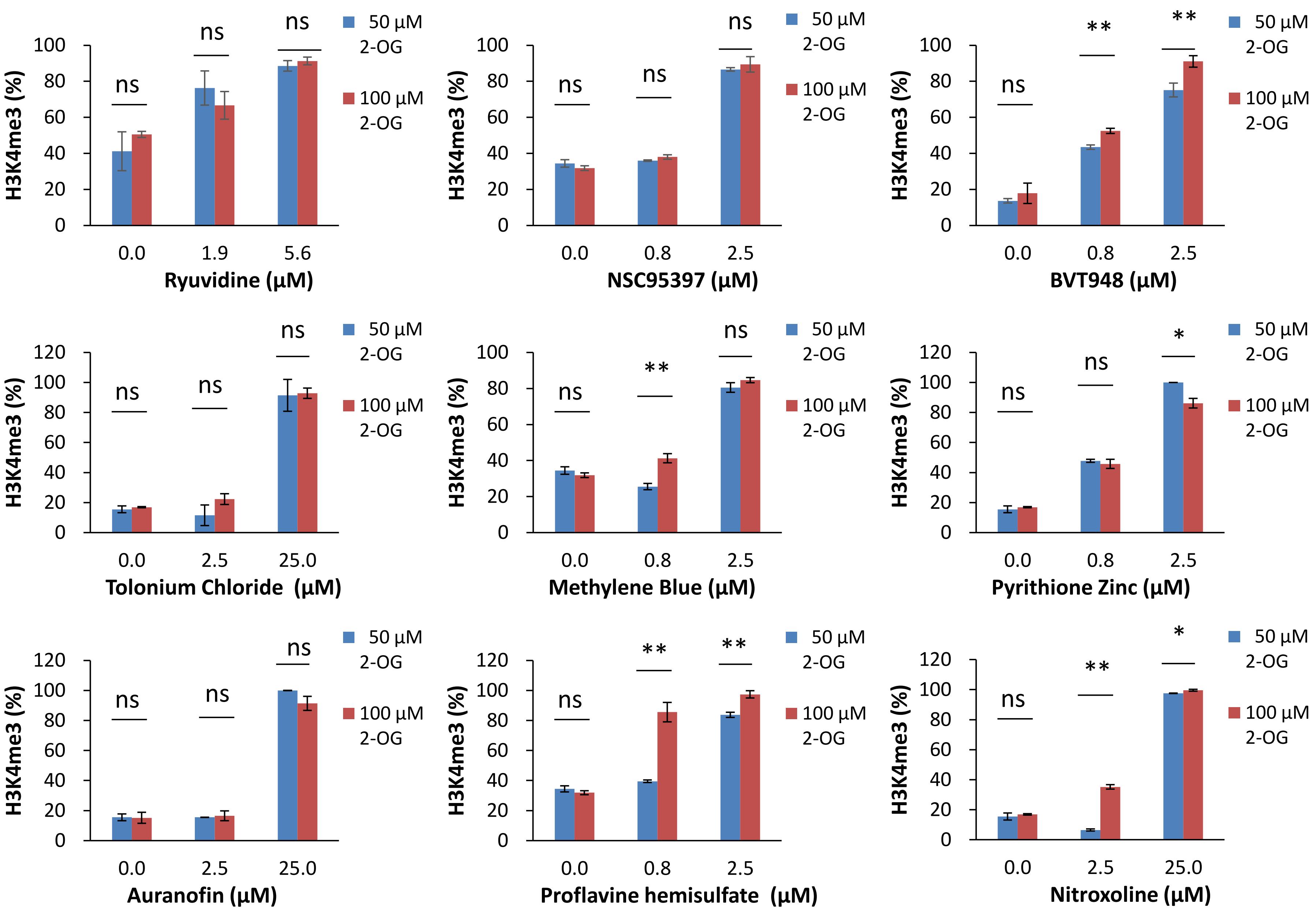

Supplementary Fig. 6

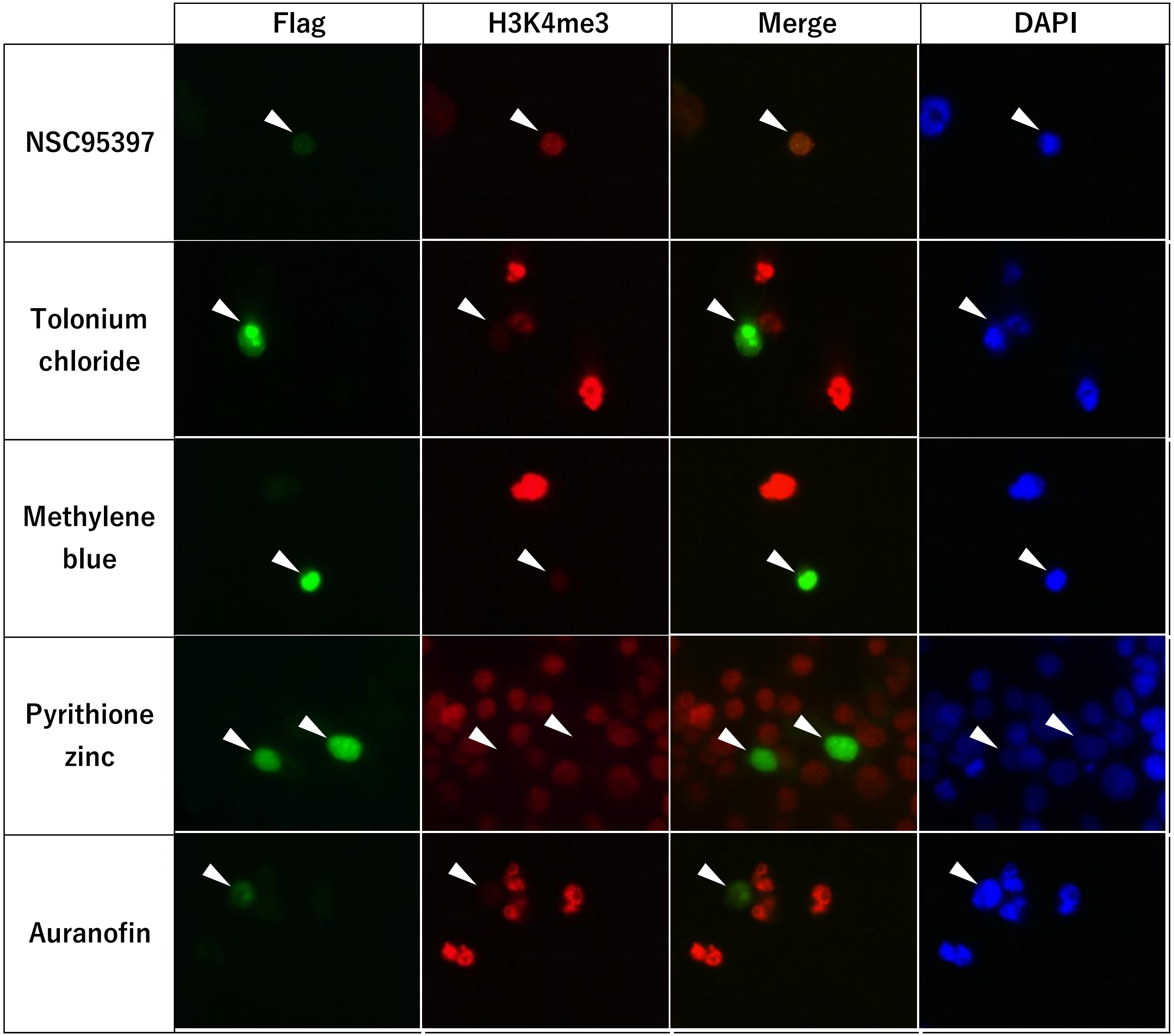

Supplementary Fig. 7

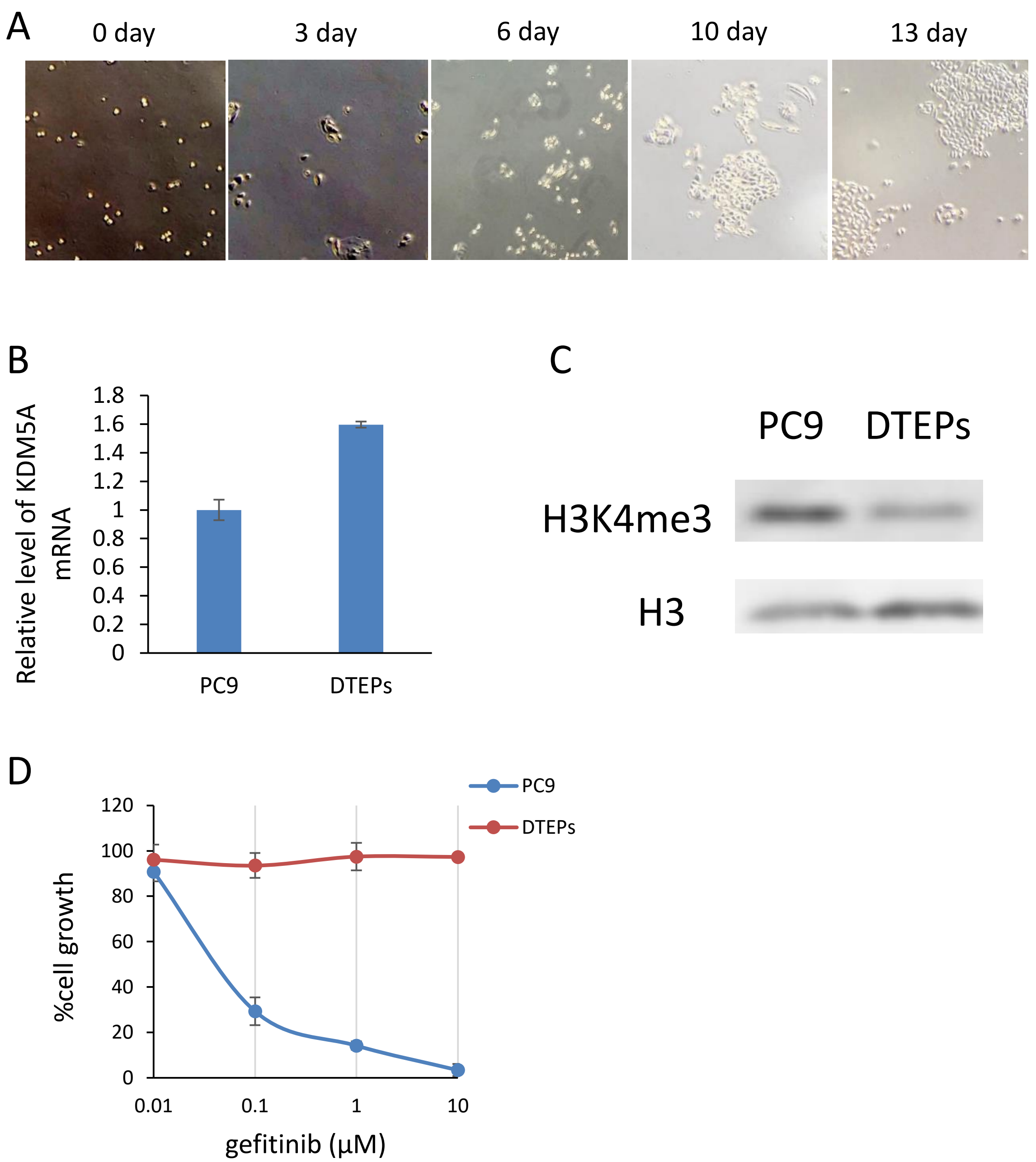

Supplementary Fig. 8

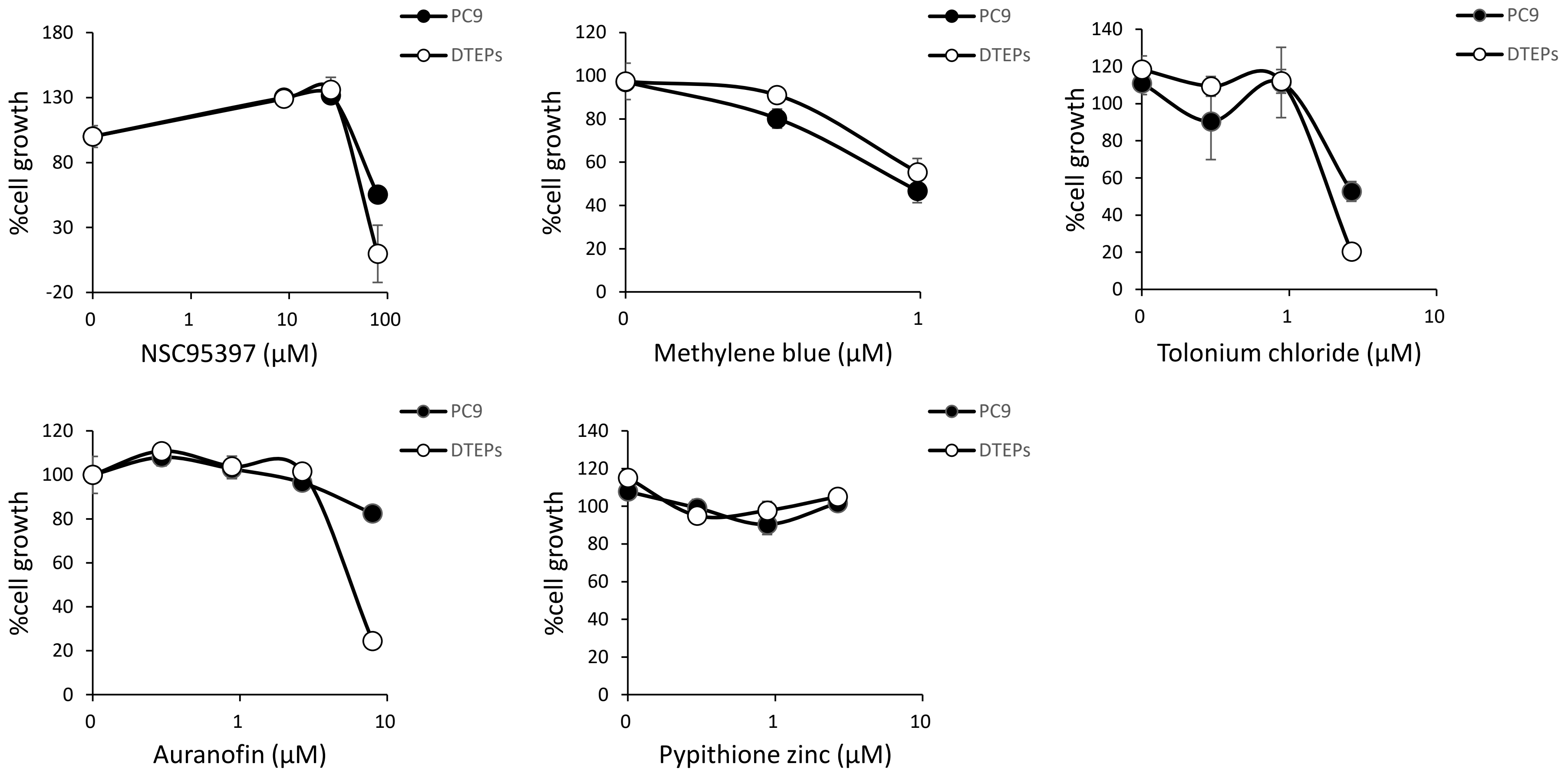

Supplementary Fig. 9

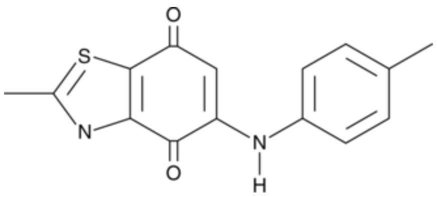

Ryuvidine

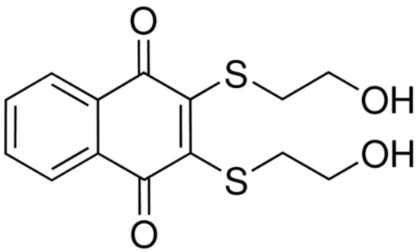

NSC95397

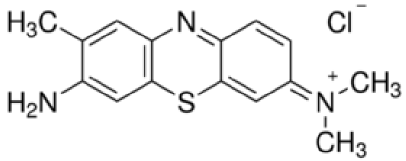

Tolonium chloride

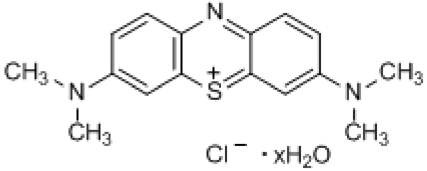

Methylene Blue

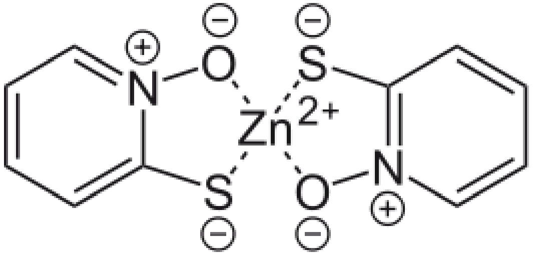

Pyrithione zinc

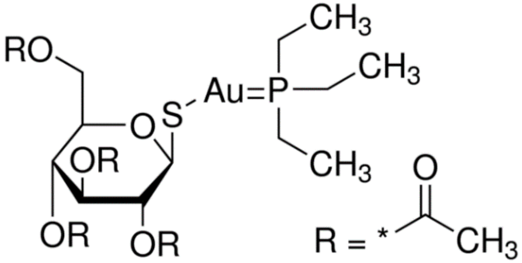

Auranofin
